# A Pixelated Microwave Near-Field Sensor for Precise Characterization of Dielectric Materials

**DOI:** 10.1038/s41598-019-49767-w

**Published:** 2019-09-16

**Authors:** Maryam Saadat-Safa, Vahid Nayyeri, Ali Ghadimi, Mohammad Soleimani, Omar M. Ramahi

**Affiliations:** 1 Antenna and Microwave Research Laboratory, Iran University of Science and Technology, Tehran, 1684613114 Iran; 2 School of Electrical Engineering, Iran University of Science and Technology, Tehran, 1684613114 Iran; 3 School of Advanced Technologies, Iran University of Science and Technology, Tehran, 1684613114 Iran; 4 Department of Electrical and Computer Engineering, University of Waterloo, Waterloo, Ontario N2L3G1 Canada

**Keywords:** Electrical and electronic engineering, Characterization and analytical techniques

## Abstract

A highly sensitive microwave near-field sensor based on electrically-small planar resonators is proposed for highly accurate characterization of dielectric materials. The proposed sensor was developed in a robust complete-cycle topology optimization procedure wherein first the sensing area was pixelated. By maximizing the sensitivity as our goal, a binary particle swarm optimization algorithm was applied to determine whether each pixel is metalized or not. The outcome of the optimization is a pixelated pattern of the resonator yielding the maximum possible sensitivity. A curve fitting method was applied to the full-wave simulation results to derive a closed form expression for extracting the dielectric constant of a chemical material from the shift in the resonance frequency of the sensor. As a proof of concept, the sensor was fabricated and used to measure the permittivity of two known liquids (cyclohexane and chloroform) and their mixtures with different volume ratios. The experimentally extracted dielectric constants were in an excellent agreement with the reference data (for pure cyclohexane and chloroform) or those obtained by mixture formulas.

## Introduction

Dielectric constant or relative permittivity is one of the most important characteristics of materials whose accurate determination is crucial in various areas such as the food industry, agriculture, medicine, health-care, and military and defense^1,2^. Accurate determination of materials needs sensing methods for characterization of dielectric materials. Methods based on RF and microwave measurements are amongst the most reliable candidates to provide accurate results^3–5^. These methods have evolved over time and mostly originated from other technologies that were intended for a completely different purpose, mainly for signal or energy transmission, such as the coaxial probe, the microstrip transmission line, the free-space propagation method, and the parallel plate waveguide. All these techniques were based on measuring the reflection coefficient with and without the material under test. The major challenge with all these methods is the device physical profile and sensitivity. The cavity resonator method provides very high accuracy and are less sensitive to noise and undesired loss and phase shift generated during measurement^6,7^. However, this method is not flexible for measuring a wide range of materials or different concentrations of different materials since different resonators need to be used.

The advent of metamaterials, which can summarily be described as an ensemble of electrically-small resonators (ESR), inspired a silent revolution in the design of sensors, material characterization techniques, and even imaging. These new class of resonators are essentially electrically-small planar resonator (ESPR) circuits with high field localization^8,9^. The strong interaction between the highly-localized field, which is considered as a near field, with the material under test (MUT) makes the sensors based on ESPR highly sensitive. While they provide higher sensitivity in comparison to the classic techniques mentioned above, their highest advantage is that they can be fabricated using printed circuit board technology, thus making them low profile, low cost, and facilitating their integration within printed circuit boards.

While the ESPR is designed to exhibit resonance at a specific frequency, its resonance phenomenon is unlike the cavity resonator where the resonance is based on the constructive interference of traveling waves (waves-based resonance). The ESPR resonance is quasi-static in the sense that the geometrical structure of the ESPR gives rise to a distributed inductance and capacitance which leads to circuit-like resonance in the sense of achieving a purely resistive input impedance. The essence of how the ESPR performs the permittivity measurement is by detecting the change that the MUT causes in the resonator’s resonance frequency and quality factor. This change happens when the MUT occupies the space of the electric field which is generated by the resonator. Earlier designs of ESPR were based on the split-ring resonator (SRR) and the complementary split-ring resonator (CSRR), simply because these designs were the classic building blocks used for metamaterials^10–22^. Later works used designs based on physical considerations such as increasing the capacitance and/or inductance to achieve improved functionality and performance^23–34^. Circuit models were also developed for predicting the sensitivity of the new sensors^35–38^. However, the circuit models were not intended as design tools but rather for validation only. The difficulty in the design emanates from the fact that the ESPR is essentially a quasi-static resonator rather than a waves-based resonator. Therefore, the maximum potential of the sensitivity for the ESPR-based sensors remains unknown.

Generally, sensitivity is the parameter that determines the capability of a sensor to sense small changes in the parameter of interest, which is the dielectric constant of the MUT in this work. Although there is an enhanced localized field in such devices, the field is mostly concentrated in the substrate (host medium) which limits the interaction between the field and the MUT, hence restricting the sensitivity of the sensor to changes in the MUT. To address this limitation, several approaches have been proposed^22,33,36–39^. For example in^22^, a channel was created inside the substrate to host the MUT, thus leading to an improved sensitivity. However, fabricating a channel inside the substrate can be challenging from a practical point of view. To improve the sensitivity, the planar resonator was assisted by an active feedback loop including a transistor amplifier^36–38^. This technique enhanced the localized electric field and thus the sensitivity significantly; however at the cost of increasing the complexity of the circuit. In another work^33^, using a 3-D parallel plate capacitor, the over capacitance of the sensor was increased which resulted in an enhancement in the stored electric energy in the sensing volume, thus improving the sensitivity. This technique, however, can increase the complexity of fabrication and the overall physical profile of the sensor. In a recent work^39^, using fractal geometries of CSRR^40^, the sensitivity of ESPR-based sensors was enhanced. However, the design of fractals does not follow a systematic and streamlined procedure.

In this paper we explore the potential of the ESPR for maximum sensitivity. Since a theoretical prediction of the sensitivity is difficult to achieve or even impossible, our approach is to optimize the surface of the ESPR for maximum sensitivity. The optimization is achieved by applying a robust, complete-cycle shape optimization procedure. The shape optimization approach is based on the pixelization of the sensing area of the sensor and then applying a binary optimization algorithm to maximize the sensitivity. This design procedure has shown high effectiveness in different technologies such as frequency selective surfaces^41^, high impedance surfaces^42^, radar cross section reduction^43^, and in electromagnetic energy harvesting and wireless power transfer^44^.

## Design Procedure

**Figure 1:**
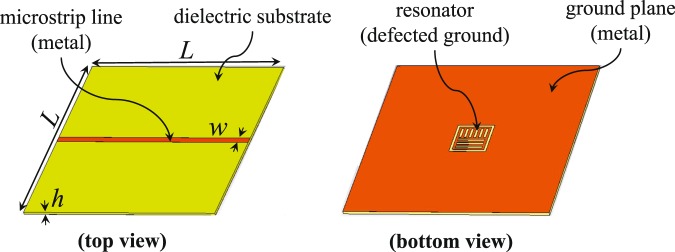
Top and bottom views of a microstrip transmission line loaded with a modified resonator based on CSRR. *L* = 50 mm, *w* = 1.1 mm, and *h* = 0.5 mm.

The fundamental mechanism of ESPR is based on quasi-static resonance which implies that a relationship between the resonance frequency, maximum sensitivity and the shape/geometry of the resonator cannot be determined analytically (as in an equation form). Therefore, our approach in this work is to find the maximum sensitivity through an effective optimization method. For this purpose, building a parametric model that can be optimized using built-in optimizers in commercial electromagnetic (EM) solvers is not possible because our optimization is not about changing the dimensions of the model but rather about its shape. The sensor that is optimized here is based on etching part of a ground plane (the sensing area) and exciting the sensor with a microstrip transmission line, as shown in Fig. 1. It should be noted that a Rogers RO4003C laminate with a dielectric constant of 3.55, a loss tangent of 0.0027, and a thickness of 0.5 mm was used as the substrate. Our optimization procedure is essentially making a decision as to which parts of the sensing area are covered with metal and which parts are not (etched). This can be achieved by pixelization of the sensing area and applying a binary optimization algorithm. A binary optimizer would assign one of two states to a specific part of the model: one state refers to metalization and the other state to no metalization. These specific parts are generated by dividing the sensing area into pixels.

**Figure 2:**
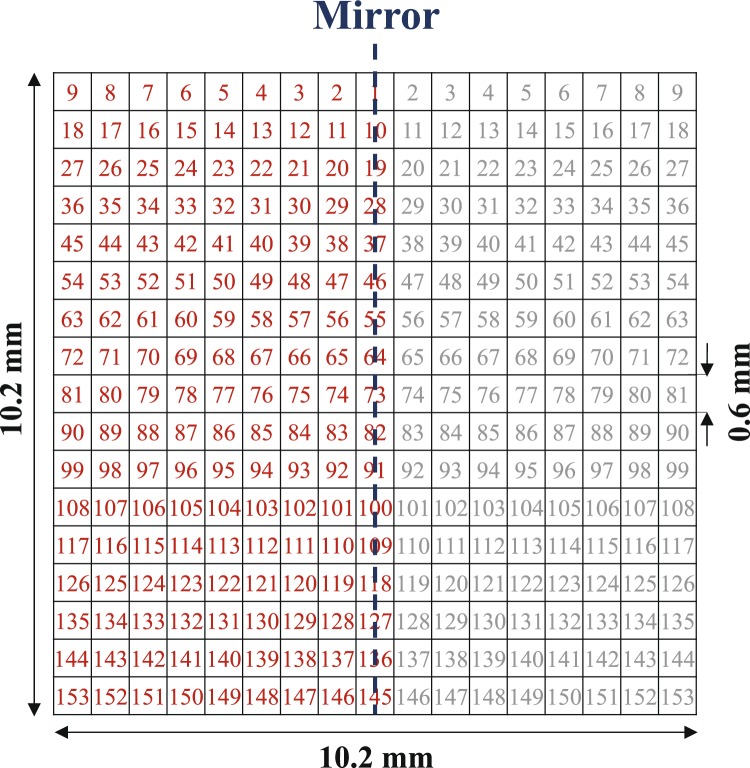
Pixelization of the sensing area.

A square of size 10.2 mm × 10.2 mm in the middle of the ground plane was considered as the sensing area and was pixelated into 17 × 17 pixels with a resolution of 0.6 mm × 0.6 mm, as shown in Fig. 2. We emphasize, however, that the sensor’s overall size is not optimized. Next, a binary value of 1 or 0 was assigned to each pixel so that each pixel was represented as a bit where having the value of 1 or 0 indicates the presence or absence of metal on the area of the pixel. In such a way, a string of bits can represent the shape of the ESPR. Therefore, a binary optimization algorithm can be applied to this string (such that each bit is an optimization parameter) for optimizing the shape of the resonator for maximum sensitivity.

**Figure 3:**
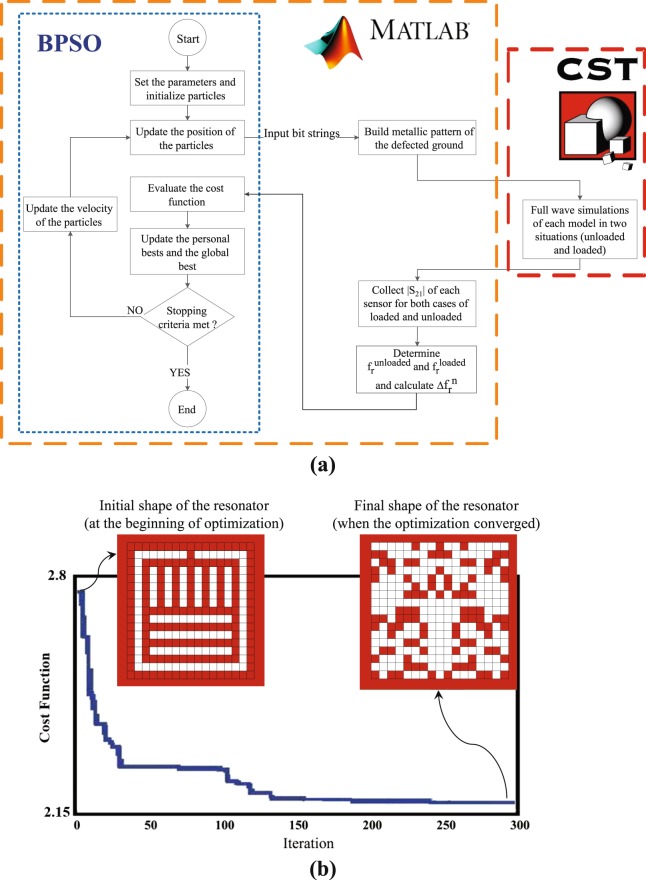
(**a**) Flowchart of the design procedure, (**b**) The value of the cost function during the iterations of the optimization algorithm; the left and right insets show the initial and final shapes of the resonator at the beginning and end of the optimization procedure, respectively.

To reduce the number of independent bits (i.e., the number of optimization parameters) and thus achieve faster convergence of the optimization algorithm, the design was constrained by enforcing a mirror symmetry with respect to an axis perpendicular to the feed microstrip line. Hence, as shown in Fig. 2, the number of the independent bits is reduced to 153. The bit numbering is also shown in the figure. Since setting initial value of the optimization parameters strongly impacts the convergence of every optimization method, to further accelerate the convergence, initial values of the bits were set such that the initial shape of the resonator was a scaled version of the one used in an earlier work^31^ which is shown in Fig. 1. A zoom-in view of the initial shape of the resonator is shown in the left inset of Fig. 3(b) wherein the pixelization is indicated. This shape corresponds to a bit string of $$[111111111100000001111111101101010101$$$$101010101101010101101010101101010101$$$$1111111010000001011111111010000001011$$$$11111101000000101111111101000000001111111111]$$, where the bit numbering is indicated in Fig. 2.

The optimization algorithm used in this work is based on particle swarm optimization (PSO)^45,46^, which is a robust global optimization method. Since each optimization parameter can have either of two states (0 or 1), a binary version of the PSO, namely the binary particle swarm optimization (BPSO) algorithm was implemented in this work^47,48^. In the PSO and BPSO, a population (called a swarm) of candidate solutions (called particles) moves around the search space such that each particle’s position is a candidate solution of the problem.

During the iterations of the algorithm, the velocity and position of each particle are updated using simple formulas (see the Appendix) such that the movement of each particle is influenced by the local best known solution which has been experienced by itself and also the global best known solution which has been found by the entire swarm. It is expected that after some iterations that depend on the dimension of the problem, the swarm moves towards (converges to) the best solution in the search space. The flowchart of the BPSO algorithm is presented in Fig. 3(a) and a brief mathematical description is provided in the Appendix.

In every optimization algorithm, a cost function is required to evaluate each candidate solution. Therefore, the optimization goal becomes the minimization of the cost function. The goal of our optimization is to maximize the sensitivity of the sensor to changes in the dielectric constant of the MUT. Since the change in the dielectric constant of the MUT is sensed by the change in the resonance frequency, when loaded with an specific sample, the higher the resonance frequency shift the loaded sensor exhibits with respect to the unloaded one, the higher the sensitivity of the sensor. Therefore, the cost function is defined as

1 $${\rm{Cost}}\,{\rm{Function}}=\frac{1}{{\Delta {\rm{f}}}_{{\rm{r}}}^{{\rm{n}}}({\varepsilon }_{{\rm{r}}}=10)}$$

where ${\Delta {\rm{f}}}_{{\rm{r}}}^{{\rm{n}}}({\varepsilon }_{{\rm{r}}}=10)$ is the normalized resonance frequency shift of the sensor when loaded with a sample having ${\varepsilon }_{{\rm{r}}}=10$. It should be noted that the normalized resonance frequency shift due to a material having ${\varepsilon }_{{\rm{r}}}=10$ is considered in the cost function. This choice is primarily because the interest in this work is characterization of materials with a dielectric constant ranging between 1 and 10, which include a wide range of chemical materials.

The process of optimizing the shape of the sensor was performed by writing a code in MATLAB^49^ and linking it to the commercial electromagnetics full-wave solve CST^50^. The diagram of this process, including the relationship between MATLAB and CST is shown in Fig. 3(a). In this process, after setting the initial parameters and generating binary strings (each consisting of 153 bits), each string is converted to a pattern which can be simulated in CST. In CST, each model (sensor) is simulated when it is unloaded and when loaded with a sample having ${\varepsilon }_{{\rm{r}}}=10$. Next, the results of the full-wave EM simulations are fed back to MATLAB. Using the received transmission coefficients, the resonance frequencies (${{\rm{f}}}_{{\rm{r}}}^{{\rm{unloaded}}}$ and ${{\rm{f}}}_{{\rm{r}}}^{{\rm{loaded}}}$) and the normalized resonance frequency shift (${\Delta {\rm{f}}}_{{\rm{r}}}^{{\rm{n}}}({\varepsilon }_{r}=10)$) are determined. Then in the loop of the BPSO algorithm, the cost function is calculated, then the personal and the global bests are updated. Next the velocity of the particles is updated, and finally the particles are assigned new updated positions.

By setting the swarm size to 100, the BPSO algorithm converged after approximately 250 iterations. Figure 3(b) shows the values of the cost function during the iterations of the algorithm indicating the decrease of the cost function from 2.78 (for the initial shape shape of the sensor) to 2.17 (for the final shape), or equivalently ${\Delta {\rm{f}}}_{{\rm{r}}}^{{\rm{n}}}({\varepsilon }_{{\rm{r}}}=10)$ was increased from 0.36 to 0.46.

The pattern of the optimized sensor is shown in the inset of Fig. 3(b), corresponding to the following bit string, $$[100100000110000011000110110100000101$$$$01001000101010000010111000000000000000011100000011101100$$$$10011010010001000$$$$00110000001000110000010001001110000010001101]$$.

## Results

**Figure 4:**
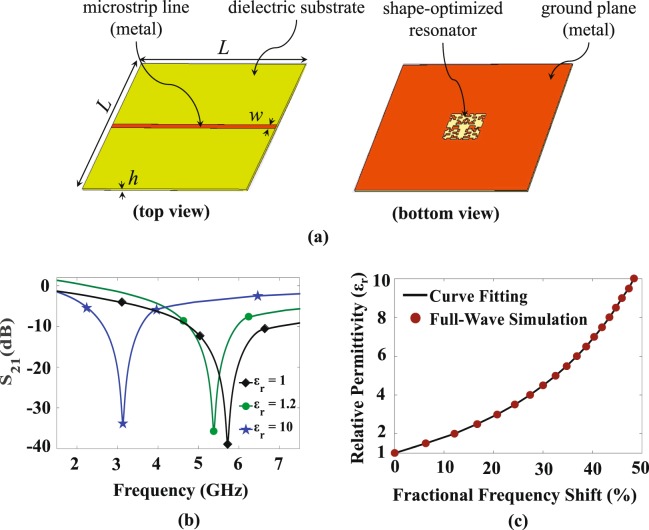
The proposed sensor with maximum sensitivity. (**a**) The top and bottom views where *L* = 50 mm, *w* = 1.1 mm, and *h* = 0.5 mm. (**b**) The transmission coefficients (|*S*_21_|) of the sensor when it is unloaded and loaded with MUTs having ${\varepsilon }_{r}=1.2$ and ${\varepsilon }_{r}=10$. (**c**) The relative permittivity of the MUT as a function of the normalized frequency shift; the markers indicate data obtained by the full-wave EM simulations and the solid line is the fitted curves.

Figure 4(a) shows a schematic of the sensor which was resulted from the optimization procedure. As shown in Fig. 4(b), this sensor shows an unloaded resonance frequency of 5.63 GHz. When the sensor is loaded with a MUT having a relative permittivity of 10, the resonance frequency shifts to 3.04 GHz, i.e., a normalized resonance frequency shift of 46%.

It is worth mentioning that the thickness of the sample impacts the sensor responses. It is expected that by increasing the sample thickness, the change in the sensor response (with respect to the unloaded sensor) is increased because the interaction between the fields and the MUT is enhanced. However, since the fields are highly localized in the proximity of the CSRR, the field interaction with the MUT saturates beyond a certain thickness; hence, the sensor response does not change considerably. According to our simulations, for the designed sensor, this thickness is around 3 mm. So, to minimize the effect of the sample thickness in the experiments, we set the thickness of the samples to 4 mm.

**Figure 5:**
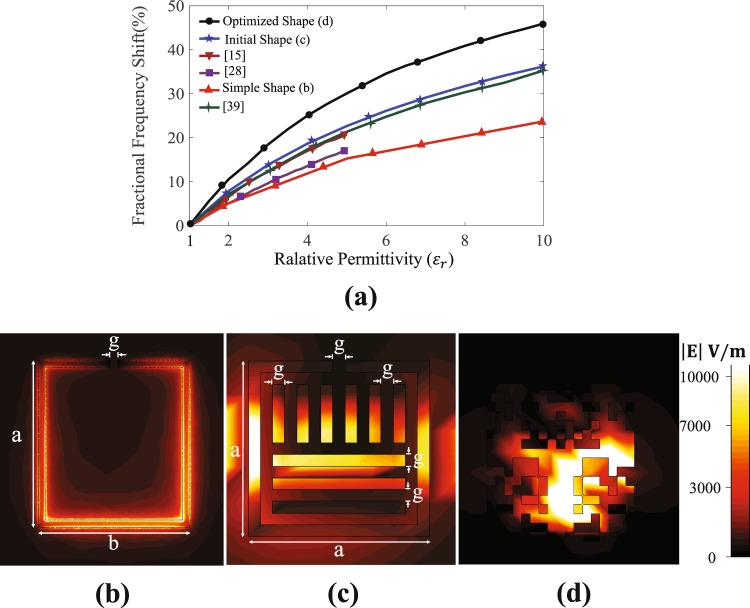
(**a**) Comparison between the sensitivity of the proposed shape-optimized sensor with that of the earlier works. (**b**) The electric field intensity distribution on the simple CSRR resonator where *a* = 8 mm, *b* = 8.6 mm, *g* = 0.4 mm. (**c**) The electric field intensity distribution on the resonator which was considered as the initial state of the optimization (shown in Fig. 1) where *a* = 9 mm, *g* = 0.6 mm. (**d**) The electric field intensity distribution on the shape-optimized resonator.

Next, by gradually increasing the relative permittivity of the MUT from 1 to 10, in Fig. 5(a), ${\Delta {\rm{f}}}_{{\rm{r}}}^{{\rm{n}}}$ of the new designed (optimized) sensor is compared with ${\Delta {\rm{f}}}_{{\rm{r}}}^{{\rm{n}}}$ of a simple CSRR sensor (see Fig. 5(b)), the modified CSRR sensor which was considered as the initial state of the optimization (shown in Fig. 1), and those recently introduced in^15,28^ and^39^. (Notice that, in^15^ and^28^, the results for ${\varepsilon }_{{\rm{r}}}$ of more than 5 were not reported. Also notice that, in^39^, several fractal sensors were designed; the simulation results of the one which was fabricated were used in our comparison). It is evident that for every dielectric constant of MUT, the optimized sensor using pixelization and shape optimization exhibits the highest ${\Delta {\rm{f}}}_{{\rm{r}}}^{{\rm{n}}}$ (see Fig. 5(a)) such that for dielectric constants of 2 and 10, this sensor shows ${\Delta {\rm{f}}}_{{\rm{r}}}^{{\rm{n}}}$s of 10% and 46% while those of the sensor of Fig. 1 (which has the second-highest ${\Delta {\rm{f}}}_{{\rm{r}}}^{{\rm{n}}}$) are 8% and 36.2%, respectively. Thus, for dielectric constants of 2 and 10, the ${\Delta {\rm{f}}}_{{\rm{r}}}^{{\rm{n}}}$ was increased 1.25 and 1.27 times, respectively.

To understand the underlying mechanism behind this improvement, Fig. 5(b–d) show a comparison between the field intensity distributions on the simple CSRR, the resonator which was considered as the initial state of the optimization, and the proposed shape-optimized resonator. From this comparison, it can be concluded that the sensitivity improvement in the pixelated sensor is due to 1) a stronger field on the sensing area such that the maximum intensity of the electric field is three times higher than that in 2(c), and 2) a larger effective sensing area wherein the electric field has a strong intensity. These two features provide broader and more effective coupling (interaction) between the sensor and the MUT.

The dielectric constant of an unknown MUT can be extracted from the shift in the resonance frequency of the loaded sensor with respect to the unloaded sensor or equivalently from the normalized resonance frequency shift. Figure 4(c) shows the relative permittivity of the MUT as a function of the normalized resonance frequency shift. In this figure, the markers indicate the results of the full-wave EM simulations. By applying curve fitting^51^, a mathematical formula was extracted giving ${\varepsilon }_{{\rm{r}}}$ of the MUT as a function of ${\Delta {\rm{f}}}_{{\rm{r}}}^{{\rm{n}}}$ as

2 $$\begin{array}{rcl}{\varepsilon }_{r} & = & 1.003+6.594\Delta {f}_{r}^{n}+46.67{(\Delta {f}_{r}^{n})}^{2}-219.6{(\Delta {f}_{r}^{n})}^{3}\\  &  & +\,579.6{(\Delta {f}_{r}^{n})}^{4}-410.5{(\Delta {f}_{r}^{n})}^{5}.\end{array}$$

As seen in Fig. 4(c), the full-wave EM simulation results give a strong agreement with the fitted curve.

**Figure 6:**
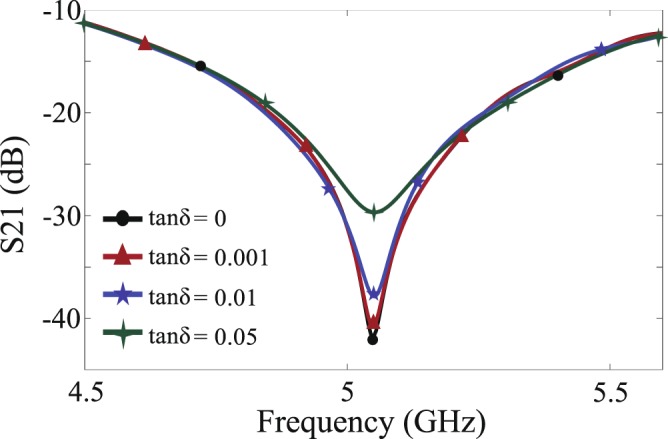
Sensor responses for lossy MUTs having ${\varepsilon }_{r}=2$ and different values of electric loss tangent.

While in our design procedure, the MUT is considered to be loss-less; our simulations showed that the designed sensor works for materials with a low and moderate loss, as well. This is due to the fact that the dielectric loss (i.e., the loss tangent) of the MUT influences the quality factor much more than the resonance frequency^29^, as shown in Fig. 6. The figure shows the responses of the sensor for different MUTs having different values of loss tangent between 0 to 0.05 but the same dielectric constant of 2. The resonance frequencies are listed in the second column of Table 1, showing a very slight change. To demonstrate the accuracy of the proposed sensor for measuring the dielectric constant of lossy materials, we retrieved the dielectric constant of the lossy samples using the resonance frequencies listed in Table 1. By applying (2), the dielectric constants of the samples were extracted, given in the third column of Table 1. It is seen that for a low-loss material with a loss tangent of 0.001, the deviation of the retrieved dielectric constant from the actual value is inappreciable; however, this deviation increases to 0.02 (i.e., 1%) for a moderately lossy material with a loss tangent of 0.05.

**Table 1:** Resonance frequencies and retrieved real permittivity when the sensor is loaded with a lossy material having a dielectric constant of 2.

Electric Loss Tangent	Resonance Frequency (GHz)	Retrieved Dielectric Constant
0	5.050	2.00
0.001	5.050	2.00
0.01	5.045	2.01
0.05	5.040	2.02

### Experimental validation

**Figure 7:**
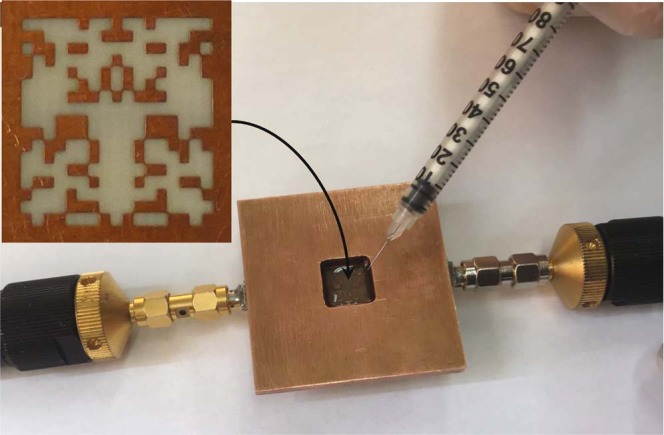
Measurement of a dielectric liquid material using the fabricated sensor and a VNA; the inset shows a zoom-in view of the pixelated pattern of the resonator which was etched on the ground plane of the microstrip line.

Using a PCB technology, the optimized sensor was fabricated by etching the ground plane of a 0.5 mm RO4003C substrate (see the inset of Fig. 7) with a 50 Ω (width = 1.1 mm) microstrip line that is used as for excitation. Notice that the design resulted in some pixels touching each other in the corners. In our simulations, these pixels are assumed to be connected to each other in a way that current can flow through the small intersection. However, in the fabrication process, these pixels can be disconnected due to corrosion by the etching liquid (photo-lithography was used). To remove this problem, the corner junctions were slightly widened as can be seen the inset of Fig. 7.

As shown in Fig. 7, a copper plate with a thickness of 4 mm and a hole in the middle was placed on the ground plane of the board. The hole is 11.8 mm × 11.8 mm, slightly larger than the sensing area, thus effectively creating a small container that includes the sensor’s area in the bottom and an open top for putting in or pouring in the MUT. Notice that since as shown in Fig. 5(d), the field is highly localized in the area of the CSRR, placing a copper plate (having a hole larger than the sensing area of the sensor) on the ground plate (where the field strength is insignificant) changes the sensor response slightly. Nevertheless, in all the simulations except the simulations during the iterations of the optimization algorithm, the effect of the plate in the sensor response was taken into account.

**Figure 8:**
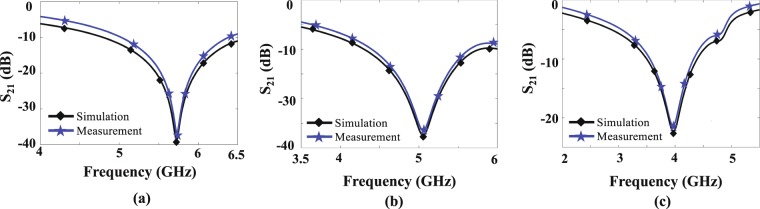
Comparisons between the simulated and measured |*S*_21_| of the sensor when it is unloaded (**a**) and is loaded with cyclohexane (**b**) and chloroform (**c**).

Two different tests were performed. In the first, the transmission coefficient (|S_21_|) of the sensor when it was unloaded and was loaded with cyclohexane (C_6_H_12_) and chloroform (CHCl_3_) having a known relative permittivity of 2.02 and 4.81, respectively^52^, was measured using a vector network analyzer (VNA). The measured and simulated transmission coefficients are seen in Fig. 8 showing a strong agreement between the simulations and experiments. Our measurements shows an unloaded resonance frequency of 5.64 GHz and loaded resonance frequencies of 5.03 GHz and 3.99 GHz for cyclohexane and chloroform, which correspond to normalized resonance frequency shifts of 0.11 and 0.29, respectively. Applying (2) to these measured data, the relative permittivity of cyclohexane and chloroform was extracted to be 2.06 and 4.79, which are very close to the reported data in^52^, i.e., 2.02 and 4.81, respectively.

In the second set of experiments, we used the fabricated sensor to extract the dielectric constant of mixtures of two materials. Mixtures of chloroform and cyclohexane with different volume ratios of 20:80, 22:78, 50:50, 75:25, and 80:20 were prepared (the first and the second numbers indicate the volume percentage of chloroform and cyclohexane, respectively). Each sample was tested with the optimized sensor and the transmission coefficient was measured. Then, the loaded resonance frequency, the normalized resonance frequency shift and subsequently, using (2), the relative permittivity of each mixture was obtained which are given in Table 2. It should be noted that Table 2 demonstrates that even when the dielectric constant of the MUT changes slightly, for example from 2.62 (for a volume fraction of 20:80) to 2.70 (for a volume fraction of 22:78), the resonance frequency changes by 40 MHz, which can be easily detected using measurement equipment.

**Table 2:** Measurements of the resonance frequency and dielectric constant of mixtures of chloroform and cyclohexane with different volume ratios.

Volume Ratio (%)	Resonance Frequency (GHz)	Measured *ε*_*r*_
0:100	5.03	2.06
20:80	4.77	2.62
22:78	4.73	2.70
50:50	4.42	3.47
75:25	4.25	3.94
80:20	4.20	4.10
100:0	3.99	4.79

In the first column of the table, the first and the second numbers indicate the volume percentage of chloroform and cyclohexane, respectively.

**Figure 9:**
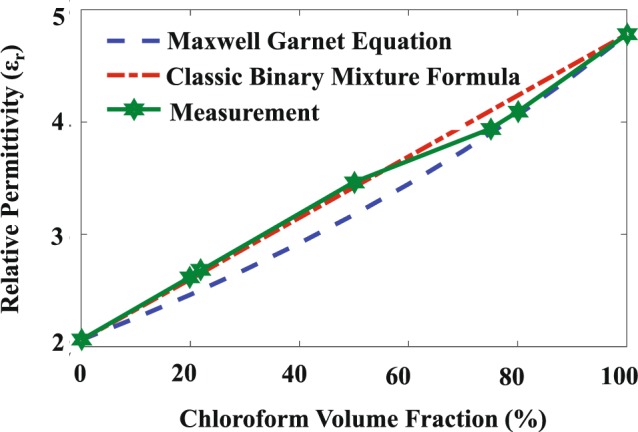
The dielectric constant of the mixture of chloroform and cyclohexane as a function of the volume fraction of chloroform.

Considering a mixture of two materials with dielectric constants of ${\varepsilon }_{1}$ and ${\varepsilon }_{2}$, there are a number of formulas to approximate the relative permittivity of the mixture. The most widely used are the classic binary mixture formula^53^

3 $${\varepsilon }_{{\rm{eff}}}={\varepsilon }_{1}{{\rm{f}}}_{1}+{\varepsilon }_{2}(1-{{\rm{f}}}_{1}),$$

and the Maxwell-Garnett approximation^54,55^

4 $${\varepsilon }_{{\rm{eff}}}={\varepsilon }_{2}+\frac{3{{\rm{f}}}_{1}\frac{({\varepsilon }_{1}-{\varepsilon }_{2}){\varepsilon }_{2}}{{\varepsilon }_{1}+2{\varepsilon }_{2}}}{1-{{\rm{f}}}_{1}\frac{({\varepsilon }_{1}-{\varepsilon }_{2})}{{\varepsilon }_{1}+2{\varepsilon }_{2}}},$$

where in (3) and (4), ${\varepsilon }_{{\rm{eff}}}$ is the effective relative permittivity of the mixture and f_1_ is the volume fraction of material 1 (therefore, the volume fraction of material 2 is $(1-{{\rm{f}}}_{1})$). By considering chloroform and cyclohexane as material 1 and 2, let us set ${\varepsilon }_{1}$ and ${\varepsilon }_{2}$ to 4.79 and 2.06, respectively. The variation of the effective permittivity of the mixture as a function of the volume fraction of chloroform (f_1_) is shown in Fig. 9. The figure presents the approximations obtained by the classic binary mixture and the Maxwell-Garnett formulas and also the results obtained by our measurements. Interestingly, for low volume fractions of chloroform, our measured results are very close to the approximation of (3), while for high volume fractions of chloroform, the measurements are close to the Maxwell-Garnett approximation (4).

## Conclusion

A highly sensitive microwave sensor for precise measuring of the permittivity of dielectric materials was presented. Inspired by a CSRR loaded line, the proposed sensor is basically a microstrip transmission line where a resonator is etched on its ground plane. Since there is a strong electric field (produced by the microstrip line) in the vicinity of the resonator (the defected ground), placing a sample of MUT on this area results in an interaction between the field and the MUT and consequently leads to change in the resonance frequency which is detected by the transmission coefficient of the line. This change in the resonance frequency which is used for sensing of the dielectric constant of the MUT determines the sensitivity of the sensor. We showed that the shape of the resonator (i.e., the pattern of the defected ground) has considerable influence on the coupling between the fields and the MUT. This coupling significantly impacts the change in the resonance frequency and thus the sensitivity of the sensor.

We designed the shape of the resonator by applying a binary optimization algorithm to a pixelated area and linking it to a full-wave EM simulator in a very systematic and fully automated approach to achieve the maximum possible sensitivity. It was shown that this highly robust design approach noticeably improved the sensitivity compared to similar designs. A curve fitting method was applied to the EM simulation results and a simple, accurate formula for extracting the permittivity in terms of the frequency shift of the sensor (with respect to the unloaded sensor) was obtained.

The designed sensor was fabricated using an inexpensive PCB technology and was experimentally tested. First the transmission coefficient of the sensor was measured when it was unloaded and loaded with two known liquids, cyclohexane and chloroform. Very strong agreement between the measured and simulated transmission coefficients validated our simulations. The re-extracted permittivity of these liquids was also in strong agreement with the reference data. In another test, mixtures of cyclohexane and chloroform with different volume ratios were prepared and tested using the fabricated sensor. The measured results showed that the fabricated sensor is capable of sensing very small changes in the dielectric constant of the MUT. The measured results were also compared with the data obtained from common formulas which estimate the dielectric constant of a mixture.

## Appendix

Here, we present a brief description of the BPSO algorithm. More details about this algorithm can be found in^47,48^. Let us assume an optimization problem with N optimization parameters. In the PSO (and its binary version, BOPS), a population (called a swarm) of candidate solutions (called particles) moves around the search space which is N-dimensional; in other words, each particle’s position is a candidate solution of the problem. Two N-dimensional vectors, namely the position vector ${X}_{m}=[{x}_{m,1},{x}_{m,2},\ldots ,{x}_{m,N}]$ and the velocity vector ${V}_{m}=[{v}_{m,1},{v}_{m,2},\ldots ,{v}_{m,N}]$ are assigned to each particle. During the iterations of the algorithms, the velocity of each particle (for example, the m^th^ particle) is adjusted according to its own experiences and the best one found by the swarm as,

5 $${V}_{m}^{t}={\rm{w}}{V}_{m}^{t-1}+{c}_{1}{e}_{1}({P}_{m}^{t-1}-{X}_{m}^{t-1})+{c}_{2}{e}_{2}({G}_{m}^{t-1}-{X}_{m}^{t-1})$$

where the superscript *t* indicates the t^th^ iteration of the algorithm, ${P}_{m}=[{p}_{m,1},{p}_{m,2},\ldots ,{p}_{m,N}]$ and $G\,=$ $[{g}_{1},{g}_{2},\ldots ,{g}_{N}]$ are the best experiences for the m^th^ particle and the swarm, respectively, *w* is the inertia coefficient, *c*_1_ and *c*_2_ are positive constants and *e*_1_ and *e*_2_ are random coefficients between 0 and 1 to guarantee the random behavior of the optimization algorithm. To further accelerates the convergence, *w* was varied from 0.9 at the beginning of the optimization to 0.4 towards the end. The suggested value for *c*_1_ and *c*_2_ is 2^48^. A limitation on the maximum velocity (V_max_) is imposed to prevent a particle from moving out of the physically meaningful solution space too often. V_max_ was suggested to be equal to the dynamic range in each dimension of the particles^48^. The initial values of particles’ velocity were set between 0 and V_max_ randomly.

While in (5), *v*_*m*,*n*_ gets real values, in the BPSO algorithm, the position vectors are binary-valued (i.e., *x*_*m*,*n*_ is 1 or 0). Therefore, to map real-valued velocities to binary-valued positions, the sigmoid limiting transformation,

6 $${\rm{S}}({{\rm{v}}}_{{\rm{m}},{\rm{n}}}^{{\rm{t}}})=\frac{1}{1+{{\rm{e}}}^{-{{\rm{v}}}_{{\rm{m}},{\rm{n}}}^{{\rm{t}}}}}$$

is commonly used. Then, the position of the m^th^ particle at $t+{1}^{th}$ iteration is updated as

7 $${{\rm{x}}}_{{\rm{m}},{\rm{n}}}^{{\rm{t}}+1}=(\begin{array}{ll}1 & {{\rm{r}}}_{{\rm{m}},{\rm{n}}}^{{\rm{t}}} < {\rm{S}}({{\rm{v}}}_{{\rm{m}},{\rm{n}}}^{{\rm{t}}})\\ 0 & {{\rm{r}}}_{{\rm{m}},{\rm{n}}}^{{\rm{t}}} > {\rm{S}}({{\rm{v}}}_{{\rm{m}},{\rm{n}}}^{{\rm{t}}})\end{array}$$

where ${r}_{m,n}^{t}$ is a random number between 0 and 1.
